# Effect of growth medium nitrogen and phosphorus on nutritional composition of *Lemna minor* (an alternative fish and poultry feed)

**DOI:** 10.1186/s12870-022-03600-1

**Published:** 2022-04-26

**Authors:** Hafiz Ullah, Bakhtiar Gul, Haroon Khan, Naveed Akhtar, Khushnood Ur Rehman, Umar Zeb

**Affiliations:** 1 Department of Botany University of Chitral, Chitral, KP Pakistan; 2 Department of Weed Science, Faculty of Crop Protection Sciences, The University of Agriculture Peshawar-Pakistan, Peshawar, Pakistan; 3 Ialamia College University, Peshawar, Pakistan; 4 Department of Biology, the University of Haripur, Haripur, Pakistan

**Keywords:** Duckweed, Lemna, Aquatic, Nutrients, Proximate composition

## Abstract

Duckweed (*Lemna minor* L.) is an aquatic macrophyte and grows profoundly on the surface of polluted water reservoirs of Pakistan. The plant can be used as a potential alternative for the fish and poultry industry to meet the promptly growing demand for feed. Our study investigates the effect of varying concentrations (ppm) of nutrients like N, P, and their combination, NP on biomass production, carbohydrate, lipid, protein, and mineral (Ca, Mg, Fe, Mn & Zn) contents of *L. minor*. The varying concentrations of N and P substantially affected the above-stated parameters. The highest biomass yield was recorded in the 30 ppm NP tank as 172 g/m^2^ day in comparison with the control tank. Higher protein, lipid, and carbohydrate contents were recorded for 30 ppm NP, 20 ppm NP, and 10 ppm NP respectively. Minerals like Ca, Mg, Fe, Mn & Zn increased in 20 ppm P and all N concentrations. The combined application of NP was more effective in boosting the protein, carbohydrate, and lipid content whereas less effective in increasing the mineral contents. A rise in the concentration of N and P showed a positive correlation with the nutritional composition of *L. minor*.

**Supplementary Information:**

The online version contains supplementary material available at 10.1186/s12870-022-03600-1.

## Introduction

Today, agriculturists are facing the challenge of an increasing feed supply for livestock, poultry, and pisciculture. To keep pace with the escalating demand for the major feedstuffs, growers are struggling for an increased supply of animal feed. The scarcity of animal feed supply has emerged as the principal matter with the expansion of the poultry and livestock industry. Obtaining sustainable and low-cost raw materials to be processed into final feed products is the key area of concern for the animal feed sector [1–3]. The rising cost of conventional animal feeds has driven agriculturists to switch to every second option of non-traditional feedstuffs. The cheaper non-conventional feed can serve as an alternative and alter the cost of the ultimate product. Animal nutritionists, therefore, have engaged themselves in finding other nonconventional nutritional resources for their animals [4].

Man has utilized and benefited from the aquatic ecosystem since time immemorial. Hydrophytes constitute the biotic component of the aquatic ecosystem which is the baseline of aquatic biodiversity. This implies that water plants are nutritionally rich and play a pivotal role to sustain the life of aquatic animals. Most of the water bodies are dominated by aquatic weeds that grow vigorously under favorable climatic conditions [5]. If not managed properly most aquatic plants may have a considerable detrimental effect on the habitat directly or indirectly [6].

Aquatic weeds are defined as the undesirable vegetation growing in water that if kept unchecked may substantially harm other aquatic biology. With progress in aquatic research, it has become evident that most aquatic plants have organic composition making them eligible to be used as animal feed. Aquatic macrophytes thus can play the role of non-conventional feed and can be utilized on a sustainable basis. Among aquatic weeds, (*Lemna minor* L*.*) is a potential candidate to be used as an economical source of animal feed in a developing country like Pakistan. The plant belongs to the family of the tiniest flowering plants, the Lemnaceae. It is one of the smallest flowering plants, rich in protein and mineral contents, and can absorb nitrogen, phosphorus, and metals efficiently from water [7]. It is a free-floating macrophyte about 3.5–10 mm in size, which thrives in the aquatic habitats of the globe. The genus Lemna is distributed among 12 species worldwide with 5 species occurring in Pakistan [8].

Globally, soyabean meal is used as a frequent source of feed because of the higher protein percentage [9]. The protein contents of *L. minor* may reach up to 45% of the dry weight [10]. It is one of the low-cost sources of minerals and pigments like beta carotene and xanthophylls [11] and contains tenfold higher of them as compared to land plants [12]. Many other research works show the use of *L. minor* as potential feed for poultry [13–15]. *L. minor* fed to 3-weeks older chicks up to 5% of mixed feed caused higher weight gain [16].

*L. minor* can multiply rapidly on the surface of stagnant water giving a smooth green appearance to the water body and adapt a broad spectrum of environmental conditions. The plant, having a short life cycle can produce several generations in a short period if favorable environmental conditions are achieved [11]. The plant can thrive and replicate maximum in water with 06–33 °C and tolerate low temperatures and hoar frost. The dry biomass yield of *L. minor* ranges between 10 and 30 tons per hectare annually with an adequate profile of dietary proteins and essential amino acids [14].

Imbalance in mineral nutrition highly retards the growth rate and multiplication of the *L. minor* plant. Variation in growth media results in the fluctuation of the proximate composition of the biomass obtained from the plant. In comparison to other growth media, *L. minor,* grown in hog wastewater (8–2%) manifested a relatively higher content of starch [17]. The contents of protein in the plant are related to the minerals in the growth medium of the plant.

As the biomass production and biochemical composition of the *L. minor* is determined by the availability and balance of nutrients in the growth medium. The present investigation is designed to determine whether a change in concentration of N and P, like other environmental factors, influences the biomass production and organic composition of the plant.

## Materials and methods

The experiment was carried out using the following materials and methods. All methods were performed under the relevant guidelines and regulations.

### Biomass cultivation

A trial was conducted in the Department of Weed Science, the University of Agriculture Peshawar, Pakistan. *L. minor* was collected from the water bodies located near the experimental site. The specimen was identified by Dr. Bakhtiar Gul. The collected plant specimen was preserved in the Herbarium of the Department of Weed and Plant Protection Sciences, the University of Agriculture Pakistan under accession number (WSW1982). The plant material was obtained from the surrounding fields was maintained in the same medium for 8 weeks without contamination and was finally used. The experiment was carried out using a Completely Randomized Design (CRD) with three replications. The plants were grown in plastic water tanks with a diameter (1m^2^) and depth of 10 in.. Fresh biomass of the plant was introduced to each tank, already amended, and labeled with N, P & NP maintained at a concentration of 10, 20 & 30 ppm each. The plants thus obtained from the plastic tanks were dried in an oven (CARBOLITE GERO-301). Analyses were made for the following proximate and mineral compositions.

### Biomass production

To evaluate the total biomass production plastic tanks of (1 m)^2^ size were filled with growth media amended with different concentrations of N, P, & NP, and a control. A triplicate for each concentration and control was used. The data was taken for 7 days of biomass increment. A 100 g fresh biomass was introduced into each container. After 7 days the biomasses were harvested and spun in a centrifugal dry (DRC 700) for 5 min to remove excess water from the fronds. Final biomass was weighed using (SF-400C digital scale). The following formula was used to calculate the total biomass production [18].

1 $$\mathrm{Tb}\left(\%\right)=\frac{\left( fb- ib\right)}{fb}\times 100$$

Where:

Tb = Total Biomass increment per (100 g) initial biomass.

fb = Final biomass of the fronds obtained from the container.

ib = Initial biomass at the time of introduction of fronds to the container.

### Protein contents

Percent nitrogen and the crude protein content were investigated by Kjeldahl’s method.

Calculations were made based on the following formula.

2 $$\mathrm{N}\left(\%\right)=\frac{\left(\mathrm{S}-\mathrm{B}\Big)\times 0.014\times \mathrm{D}\right)}{\mathrm{Weight}\left(\mathrm{S}\mathrm{ample}\right)\times \mathrm{V}}\times 100$$

Where:

N = Percentage of Nitrogen.

S = Standard acid volume used in titration (ml).

B = Volume of standard acid used in blank titration (ml).

D = Sample dilution after digestion (ml).

^*^0.014 is the milliequivalent weight of nitrogen.

### Lipid contents

The contents of lipid were analyzed through the Soxtec™ 8000. A 0.5 g sample was weighed to undergo wet digestion using petroleum ether. To heat the samples electrical heating plates were used. The samples were boiled and rinsed to recover the fat. The flasks containing the fat were weighed to measure the total lipid of the sample. The following formula was used to calculate the percentage of extractable fat.

3 $$\mathrm{Fat}\ \left(\mathrm{Extractable}\right)\%=\frac{\mathrm{W}3-\mathrm{W}2}{\mathrm{W}1}\times 100$$

Where:

W1 = Weight of sample taken for extraction (g).

W2 = Empty weight of flask (g).

W3 = Weight of flask with fat (g).

### Carbohydrate contents

The carbohydrate contents of the samples were calculated using the following formula.

4 $$\mathrm{Carbohydrate}\left(\%\right)=100-\left(\mathrm{Lipid}+\mathrm{Protein}+\mathrm{Mineral}\right)$$

### Mineral contents

Atomic Absorption Spectrometry was used to detect the mineral contents. The samples were digested through the wet digestion method using perchloric acid as solvent. The digested samples were filtered in glass filters and diluted with distilled water to make a 100 ml volume [19]. Calculations were made based on the following formula.

5 $$\mathrm{Mineral}\left(\frac{\mathrm{mg}}{100\ \mathrm{g}}\right)=\frac{\mathrm{AAS}\ \mathrm{result}\times \mathrm{V}\times \mathrm{DF}}{\mathrm{W}}$$

Where:

AAS = Atomic absorption spectrometry result.

V = Volume of sample.

DF = Dilution Factor.

W = Weight of sample.

### Data analysis

Data obtained was analyzed using Mstat-C GenStat software for ANOVA and LSD test at a 0.05 level of probability [20].

## Results

The findings of this study suggest that nitrogen and phosphorus, like other environmental factors, affect the percentage biochemical composition of (*Lemna minor*). All the parameters showed variation with changes in the concentration of nutrients supplied to the growth medium.

### Biomass increment

The effect of N and P treatment in different concentrations (ppm) showed a pronounced effect on fresh biomass of *L. minor* as compared to control. The highest fresh biomass yield was recorded in the 30 ppm NP tank as 172 g/m^2^ day. In the control tank, the average fresh biomass yield was 113.8 /m^2^ day. With the increase in the concentration of N and P, the biomass yield increased gradually. The combined treatment of NP resulted in higher biomass production than the individual application of N and P. A considerable variation in biomass yield as reported by different workers. An increment of biomass at the rate of 5.5 g/m^2^ day, 13.8 g/m^2^ day, and 15.1 g/m^2^ day have been reported by different workers [21–23]. In *L. gibba* L. the biomass increment ranged between 132.6 g/m^2^ day to 200.95 g/m^2^ day respectively [18]. For maximum biomass production, sufficient nutrient levels should be maintained [10].

### Proteins

Protein contents markedly increased in plants grown in 30 ppm NP concentration. The highest percentage of protein obtained in this treatment was 33.0 g/100 g as shown in Table 1. The lowest 27/100 g was obtained from the control. With an increase in nitrogen concentration, protein contents increased markedly. A 20 ppm increase in nitrogen concentration elevated protein contents by 2 g/100 g. A similar rise in protein contents was also observed with an increase in P concentration. The result of the collective application of NP was more significant than the independent application of N and P.

**Table 1:** Effect of Growth Medium N, P, and NP concentrations on Protein, Lipid, and Carbohydrate Contents of *L. minor*

Nutrients (ppm)	Proximate Nutritional Composition (g/100 g)		
	Protein	Lipid	Carbohydrate
10 N	27.6 ± 0.8^**ef**^	4.67 ± 1.0^f^	54.6 ± 1.0^**d**^
20 N	28.7 ± 0.9^**de**^	5.67 ± 2.0^**e**^	56.0 ± 1.5^**c**^
30 N	29.0 ± 0.8^**de**^	6.00 ± 1.5^**e**^	55.6 ± 1.5^**cd**^
10 P	29.3 ± 0.5^**d**^	6.67 ± 1.5^**d**^	57.0 ± 1.0^**bc**^
20 P	30.0 ± 0.7^**cd**^	7.00 ± 2.0^**d**^	57.6 ± 0.5^**ab**^
30 P	31.0 ± 2.0^**bc**^	8.00 ± 0.8^**c**^	58.3 ± 2.0^**ab**^
10 NP	31.0 ± 0.8^**bc**^	8.00 ± 0.9^**c**^	59.0 ± 1.7^**ab**^
20 NP	32.0 ± 1.5^**ab**^	9.00 ± 0.5^**b**^	59.3 ± 1.8^**ab**^
30 NP	33.0 ± 2.0^**a**^	10.0 ± 1.0^**a**^	60.0 ± 1.0^**a**^
Control	27.0 ± 0.5^**f**^	4.67 ± 0.5^**f**^	52.3 ± 1.0^**e**^
LSD (*p* ≤ 0.05)	1.61	0.622	3.84

Means in the table with different superscripts are significantly different (*p* ≤ 0.05) using the LSD test

### Lipids

A significant effect of the applied growth medium nutrients is depicted in Table 1. The content of lipid, 10 mg/100 g, as the highest was recorded in plants grown in 30 ppm NP medium. This value was followed by 10 mg/100 g lipid contents for plants grown in the 20 ppm NP medium. Statistical analysis of the data regarding lipid content as depicted in (Table 1) revealed that variation in nutrients of the growth medium had a significant effect on the lipid content of *L. minor*. The lowest value of 4.67 g/100 g lipid was noted in plants growing in 10 ppm N medium and control.

### Carbohydrates

The results of the contents of carbohydrates are shown in Table 1. A maximum level of carbohydrates, 60 g/100 g dry weight, was observed in the plants grown in 30 ppm NP medium and 59 g/100 g for 20 ppm respectively. The control group showed the minimum (52 g/100 g) level of carbohydrates. Individual application of N and P at the rate of 30 ppm enhanced the carbohydrate content by (2 g/100 g).

### Minerals

Five elements, Ca, Mg, Fe, Mn, and Zn showed the following results with the change in the level of N, P, and NP. The results are shown in Table 2.

**Table 2:** Elemental composition of *Lemna minor* after treatment with variable N and P combinations

Nutrients (ppm)	Mineral Composition (mg/100 g) Dry Weight				
	Ca	Mg	Fe	Mn	Zn
10 N	35 ± 0.03^**bc**^	27 ± 0.03^**fg**^	25 ± 0.41^**bc**^	3.2 ± 0.05 cd	0.07 ± 0.05c
20 N	38 ± 0.02^**b**^	28 ± 0.03^**efg**^	33 ± 0.35^**a**^	2.4 ± 0.15d	0.03 ± 0.01b
30 N	40 ± 0.25^**a**^	28 ± 0.03^**def**^	36 ± 0.55^**a**^	3.2 ± 0.40 cd	0.05 ± 0.02a
10 P	26 ± 0.05^**cd**^	29 ± 0.03^**cdef**^	23 ± 0.49^**c**^	0.7 ± 0.03 cd	0.04 ± 0.03d
20 P	26 ± 0.45^**d**^	30 ± 0.03^**bcde**^	34 ± 0.65^a^	3.2 ± 0.25d	0.06 ± 0.01a
30 P	30 ± 0.70^**a**^	31 ± 0.03^**abcd**^	35 ± 0.47^**a**^	0.8 ± 0.20 cd	0.04 ± 0.01 cd
10 NP	26 ± 0.09^**cd**^	32 ± 0.03^**abc**^	34 ± 0.75^**a**^	3.7 ± 0.18a	0.06 ± 0.05 cd
20 NP	24 ± 0.08^**d**^	32 ± 0.03^**ab**^	35 ± 0.65^**a**^	2.5 ± 0.32c	0.08 ± 0.02c
30 NP	24 ± 0.02^**d**^	33 ± 0.03^**a**^	36 ± 0.40^**a**^	2.8 ± 0.01c	0.06 ± 0.10 cd
Control	32 ± 0.09^**bcd**^	25 ± 0.03^**g**^	26 ± 0.55^**b**^	0.7 ± 0.41b	0.04 ± 0.01 cd
LSD (*p* ≤ 0.05)	0.8	0.07	0.15	0.08	0.05

Means in the above with different superscripts were significantly different (*p* ≤ 0.05) using the LSD test. The superscripts with different letters show that the means are significantly different from each other

#### Ca

Analysis of the data manifested a significant effect of altering nutrient concentrations on the elemental composition of Ca. With an increase in nitrogen concentration, a gradual increase in Ca content resulted. Phosphorus concentration of 30 ppm has a maximum increasing effect (30 mg/100 g) in calcium content while the combined effect of NP has a similar effect at all levels of concentrations.

#### Mg

A maximum of (33 mg/100 g) Mg content was observed for plants grown in 30 ppm NP followed by (32 mg/100 g) for plants grown in 20 ppm NP. Thus, Mg content was fortified by the combined application of N and P. The increase in the concentration of N increased the quantity of Mg at the same time increase in P concentration to 30 ppm also boosted the percentage of the element. In all cases, whether the two nutrients N & P are applied individually or in combination increase the total of Mg in the biomass of the plant.

#### Fe

The maximum level of Fe (36 mg/100 g) was recorded for plants growing in the 30 ppm N and 30 ppm NP as shown in Table 2. The lowest content of Fe was recorded for 10 ppm N which is 25 mg/100 g. The result shows a significant relationship between the treatments and Fe contents.

#### Mn

The fraction of Mn varied with the change in the nutrient levels of the growth medium. A higher value of the element (3.7 mg/100 g) was noted in the treatment of 10 ppm NP. Independent application of N and P also showed a significant effect. The minimum value of (0.7 mg/100 g) was depicted for control.

#### Zn

The composition of zinc is depicted in Table 2. The maximum concentration of zinc 0.082 mg/100 g) was obtained for plants grown in the medium of 20 ppm NP. The independent application of N and P also showed a significant change, but the more pronounced effect was observed in the combined application of NP.

## Discussion

The protein moiety of the *L. minor* directed a significant increase with the combined application of NP. Both nitrogen and phosphorus added to the growth medium improved protein contents in the plant. The crude proteins of *L. minor* grown in different levels of the nutrient solution may increase from (7–45%) of the dry biomass depending on nitrogen availability [10]. Nutrient deficiency, slow growth has been reported to result in protein level as low as (7%) of dry matter [24].

Lipid contents also increased with the application of N and P. For most of the investigators the effect of N, P application has resulted in no significant change in the total lipid content accumulation but only a change in the proportion of different fatty acids has resulted [25]. Both N and P are important constituents of biochemical machinery and cellular metabolism. Nitrogen being a part of chlorophyll brings a major change in photosynthetic activity when deficient. Unfertilized lagoon growing duckweeds produce as many as (4.4%) of lipid of total dry mass [14]. An increase in nutrient availability and continuous exposure to sunlight increase the growth of the plant and ultimately increased the macromolecular composition.

Our study suggests an increase in carbohydrate components with the increase of both N and P. Both elements act as growth control factors since these are key elements in the biochemistry of every plant. It was suggested that N enrichment to *Chaetomorpha linum* causes an increase in chlorophyll content of the plant, and hence more carbon fixation results [26]. Phosphorus has no direct effect on chlorophyll contents but an indirect effect on carbohydrate metabolism. It is a component of sugar-phosphate intermediates of both photosynthesis and respiration. Also, it is a part of all those reactions which involve ATP and is actively involved in the assimilation of photosynthetic products.

Phosphorus application increases the uptake of Ca in plants. It was shown that Ca uptake was higher in (*Apium graveolens* L.) after the application of P [27]. Plant cells can accumulate nutrients at much higher concentrations than are present in their root zone. This allows roots to extract nutrients from the soil solution where they are present in very low concentrations. Mobility of nutrients within the plant depends largely upon transport through cell membranes, which requires energy to transport against the concentration gradient. Here again, ATP and other high-energy phosphate compounds provide the needed energy. Calcium uptake in the root is to a large extent genetically controlled and is relatively less affected by Ca supply to the root medium, if Ca availability is adequate for normal growth [28].

The importance of magnesium cannot be negated in plant growth and development. Besides the role of Mg in chlorophyll structure and as an enzyme cofactor, another essential role of Mg in plants is in the export of photosynthates, which when impaired culminates in enhanced degradation of chlorophyll in Mg deficient leaves, resulting in higher oxygenase activity of RuBP carboxylase [29].

Most plant species rely on reduction-based Fe uptake, and much progress has been made in the past few years concerning hormonal, molecular, and whole-plant aspects of its regulation, this review focuses on the Strategy I-type mechanism of Fe acquisition. There are several other recent reviews covering iron uptake in yeast [30].

Trace element like Mn is available for biological absorption at moderately acidic pH [31, 32]. Therefore, the absorption of Mn is regulated by the presence of phosphate in the root zone. The absorption of Zn is much dependent on the pH factor. It has been observed that the absorption of micronutrients increases with low pH. Low pH coupled with mild redox increases the availability of micronutrients.

## Conclusion

The effect of both N and P was significant on the biomass production, crop growth, protein, lipid, carbohydrate, and mineral contents of *L. minor*. Literature review shows that the proximate composition of the plant showed that it contains a fair quantity of all nutrients like proteins, essential amino acids, lipids, carbohydrates, and minerals. Due to its rapid growth, it produces a lot of biomasses, and its short life cycle provides us an opportunity to be harvested twice a week. Its chemical composition is sensitive to the growing conditions and its biochemical contents like proteins, minerals, and carbohydrates can be manipulated by the nutritional status of the growth media. According to this research, *L. minor* can be grown in large tanks to harvest maximum biomass that is yet to be practiced in Pakistan. The nutritional composition of the biomass can be improved by adjusting the nutrient concentration of the aquatic medium. The nutritionally rich biomass can thus be used as feed.

## Supplementary Information

**Additional file 1.**

## Data Availability

The crude data is available and uploaded as a supplementary material file to the journal submission system.
