# Supplementary material for: Effect of growth medium nitrogen and phosphorus on nutritional composition of Lemna minor (an alternative fish and poultry feed)
Source: BMC Plant Biol. 2022 Apr 26;22:214. doi: 10.1186/s12870-022-03600-1 (PMC9040223; doi:10.1186/s12870-022-03600-1)
Supplement: Supplementary file 1 — Additional file 1. [file 12870_2022_3600_MOESM1_ESM.docx]

**EXPERIMENT- 1**

**B1: Original replicated data for the effect of (N), (P) and (NP) levels on for fresh biomass, dry biomass and growth of Duckweed (*L. minor*)**

| **Replication** | **treatment** | **Fresh biomass(gm^-2^)** | **Dry biomass(gm^-2^)** | **Crop growth (%)** |
| --- | --- | --- | --- | --- |
| R_1_ | 1 | 800 | 38 | 60 |
|  | 2 | 860 | 40.5 | 65 |
|  | 3 | 925 | 41 | 55 |
|  | 4 | 845 | 41.5 | 59 |
|  | 5 | 865 | 42 | 73 |
|  | 6 | 930 | 42.5 | 23 |
|  | 7 | 955 | 43.5 | 86 |
|  | 8 | 981.5 | 44.5 | 53 |
|  | 9 | 1030 | 45.5 | 60 |
|  | 10 | 775 | 37.5 | 60 |
| R_2_ | 1 | 795 | 40 | 50 |
|  | 2 | 825 | 40.5 | 52 |
|  | 3 | 845 | 41.5 | 50 |
|  | 4 | 865 | 42 | 63 |
|  | 5 | 920 | 42.5 | 74 |
|  | 6 | 940 | 43 | 32 |
|  | 7 | 945 | 43.5 | 61 |
|  | 8 | 1045 | 44 | 59 |
|  | 9 | 1060 | 44.5 | 62 |
|  | 10 | 802 | 38 | 70 |
| R_3_ | 1 | 840 | 41.5 | 48 |
|  | 2 | 870 | 41.5 | 44 |
|  | 3 | 905 | 42 | 44 |
|  | 4 | 926 | 42.5 | 57 |
|  | 5 | 947 | 42.5 | 68 |
|  | 6 | 952 | 43 | 43 |
|  | 7 | 965 | 43 | 66 |
|  | 8 | 985 | 43.3 | 57 |
|  | 9 | 1025 | 44 | 80 |
|  | 10 | 815 | 37 | 73 |

**B2 : Original replicated data for the effect of (N), (P) and (NP) levels on Protein, lipid,** **carbohydrate and mineral contents**

| **Replication** | **Treatment** | **Protein (%)** | **Lipid (%)** | **Carbohydrate (%)** | **Mineral (%)** |
| --- | --- | --- | --- | --- | --- |
| R_1_ | 1 | 27 | 4 | 53 | 0.08527 |
|  | 2 | 28 | 5 | 55 | 0.09833 |
|  | 3 | 28 | 6 | 56 | 0.12015 |
|  | 4 | 28 | 6 | 58 | 0.07456 |
|  | 5 | 29 | 7 | 59 | 0.11414 |
|  | 6 | 30 | 8 | 60 | 0.08286 |
|  | 7 | 30 | 8 | 61 | 0.08943 |
|  | 8 | 31 | 9 | 61 | 0.08848 |
|  | 9 | 32 | 10 | 62 | 0.08488 |
|  | 10 | 26 | 4 | 52 | 0.08431 |
| R_2_ | 1 | 28 | 5 | 55 | 0.09838 |
|  | 2 | 29 | 6 | 56 | 0.10844 |
|  | 3 | 30 | 6 | 54 | 0.11936 |
|  | 4 | 30 | 7 | 55 | 0.08576 |
|  | 5 | 31 | 7 | 56 | 0.12425 |
|  | 6 | 32 | 8 | 56 | 0.08996 |
|  | 7 | 31 | 8 | 57 | 0.09351 |
|  | 8 | 32 | 9 | 57 | 0.09159 |
|  | 9 | 33 | 10 | 58 | 0.08399 |
|  | 10 | 27 | 5 | 52 | 0.0873 |
| R_3_ | 1 | 28 | 5 | 56 | 0.09726 |
|  | 2 | 29 | 6 | 57 | 0.10954 |
|  | 3 | 29 | 6 | 57 | 0.11326 |
|  | 4 | 30 | 7 | 58 | 0.08598 |
|  | 5 | 30 | 7 | 58 | 0.12234 |
|  | 6 | 31 | 8 | 59 | 0.09486 |
|  | 7 | 32 | 8 | 59 | 0.09062 |
|  | 8 | 33 | 9 | 60 | 0.09868 |
|  | 9 | 34 | 10 | 60 | 0.09688 |
|  | 10 | 28 | 5 | 53 | 0.0835 |

**EXPERIMENT- 2**

**B3: Original replicated data for** **effect of pH levels on fresh biomass, dry biomass and crop growth of Duckweed (*L. minor*)**

| **Replication** | **Treatment** | **Fresh biomass(gm^-2^)** | **Dry biomass(gm^-2^)** | **Growth (%)** |
| --- | --- | --- | --- | --- |
| R_1_ | 1 | 625 | 40 | 25 |
|  | 2 | 690 | 44 | 38 |
|  | 3 | 825 | 46.5 | 65 |
|  | 4 | 827.5 | 43.65 | 66 |
|  | 5 | 895 | 45.9 | 90 |
|  | 6 | 715 | 39.5 | 43 |
|  | 7 | 745 | 44.2 | 49 |
| R_2_ | 1 | 590 | 37 | 35 |
|  | 2 | 675 | 41.5 | 79 |
|  | 3 | 895 | 55 | 98 |
|  | 4 | 989 | 49.5 | 80 |
|  | 5 | 900 | 40.8 | 68 |
|  | 6 | 840 | 39.3 | 57 |
|  | 7 | 575 | 35 | 15 |
| R_3_ | 1 | 690 | 44.2 | 13 |
|  | 2 | 565 | 35 | 83 |
|  | 3 | 915 | 62 | 85 |
|  | 4 | 927 | 44.5 | 66 |
|  | 5 | 830 | 46.5 | 99 |
|  | 6 | 725 | 44.3 | 45 |
|  | 7 | 610 | 37 | 22 |

**B4: Original replicated data for the effect of pH on protein, lipid, carbohydrate and mineral contents of Duckweed (*L. minor*)**

| **Replication** | **Treatment** | **Protein (%)** | **Lipid (%)** | **Carbohydrate (%)** | **Mineral (%)** |
| --- | --- | --- | --- | --- | --- |
| R_1_ | 1 | 23 | 7 | 60 | 0.04356 |
|  | 2 | 28 | 7 | 55 | 0.08055 |
|  | 3 | 31 | 7 | 51 | 0.05496 |
|  | 4 | 33 | 8 | 49 | 0.08119 |
|  | 5 | 30 | 8 | 52 | 0.09411 |
|  | 6 | 28 | 6 | 56 | 0.07922 |
|  | 7 | 19 | 2 | 53 | 0.07909 |
| R_2_ | 1 | 23 | 6.5 | 60 | 0.04486 |
|  | 2 | 27 | 7 | 56 | 0.08785 |
|  | 3 | 29 | 8 | 53 | 0.06436 |
|  | 4 | 34 | 7 | 49 | 0.08916 |
|  | 5 | 29 | 8 | 53 | 0.08411 |
|  | 6 | 26 | 6 | 58 | 0.08432 |
|  | 7 | 24 | 3 | 49 | 0.08112 |
| R_3_ | 1 | 25 | 6 | 59 | 0.04258 |
|  | 2 | 26 | 6.4 | 57 | 0.08575 |
|  | 3 | 28 | 7.8 | 55 | 0.05496 |
|  | 4 | 32 | 8 | 58 | 0.08521 |
|  | 5 | 30 | 9 | 51 | 0.09609 |
|  | 6 | 25 | 5 | 51 | 0.08945 |
|  | 7 | 22 | 3 | 50 | 0.09311 |

**EXPERIMENT- 2**

**B5: Original replicated data for the effect of salinity on fresh biomass, dry biomass and growth of Duckweed (*L. minor*)**

| **Replication** | **Treatment** | **Fresh biomass(gm^-2^)** | **Dry biomass(gm^-2^)** | **Crop growth (%)** |
| --- | --- | --- | --- | --- |
| R_1_ | 1 | 800 | 40 | 60 |
|  | 2 | 930 | 42.2 | 86 |
|  | 3 | 800 | 40.5 | 60 |
|  | 4 | 812.5 | 42.45 | 62.5 |
|  | 5 | 725 | 35 | 45 |
|  | 6 | 815 | 34 | 63 |
|  | 7 | 950 | 61.5 | 90 |
| R_2_ | 1 | 902.5 | 45.5 | 97.8 |
|  | 2 | 900 | 45 | 80 |
|  | 3 | 840 | 43.5 | 68 |
|  | 4 | 753 | 39 | 80 |
|  | 5 | 651 | 32.5 | 30 |
|  | 6 | 795 | 30.7 | 59 |
|  | 7 | 950 | 51 | 60.4 |
| R_3_ | 1 | 895 | 40 | 65 |
|  | 2 | 901 | 44.5 | 80 |
|  | 3 | 789 | 40.5 | 57.9 |
|  | 4 | 780 | 42.5 | 56 |
|  | 5 | 835 | 39.5 | 47 |
|  | 6 | 725 | 40 | 65 |
|  | 7 | 987 | 51.5 | 77 |

**B6: Original replicated data for the effect of salinity on protein, lipid, carbohydrate, and mineral contents of Duckweed (*L. minor*)**

| **Replication** | **Treatment** | **Protein (%)** | **Lipid (%)** | **Carbohydrate (%)** | **Mineral (%)** |
| --- | --- | --- | --- | --- | --- |
| R_1_ | 1 | 25 | 4 | 61 | 0.09088 |
|  | 2 | 24 | 3 | 63 | 0.06353 |
|  | 3 | 26 | 4 | 60 | 0.06377 |
|  | 4 | 27 | 3 | 60 | 0.06436 |
|  | 5 | 36 | 2 | 52 | 0.05831 |
|  | 6 | 34 | 8 | 52 | 0.07985 |
|  | 7 | 28 | 5 | 57 | 0.08769 |
| R_2_ | 1 | 26 | 6 | 58 | 0.09398 |
|  | 2 | 25 | 5 | 60 | 0.06552 |
|  | 3 | 27 | 6 | 57 | 0.06596 |
|  | 4 | 30 | 5 | 55 | 0.06756 |
|  | 5 | 32 | 3 | 55 | 0.06032 |
|  | 6 | 32 | 7 | 51 | 0.08375 |
|  | 7 | 23 | 4 | 63 | 0.08969 |
| R_3_ | 1 | 24 | 6 | 62 | 0.09498 |
|  | 2 | 26 | 4 | 60 | 0.06655 |
|  | 3 | 28 | 4 | 58 | 0.05997 |
|  | 4 | 32 | 5 | 54 | 0.06646 |
|  | 5 | 34 | 3 | 53 | 0.0633 |
|  | 6 | 33 | 7 | 50 | 0.08735 |
|  | 7 | 26 | 6 | 58 | 0.09859 |
